# In Vitro Studies Reveal Antiurolithic Effect of Antioxidant Sulfated Polysaccharides from the Green Seaweed *Caulerpa cupressoides* var *flabellata*

**DOI:** 10.3390/md17060326

**Published:** 2019-06-01

**Authors:** Dayanne Lopes Gomes, Karoline Rachel Teodosio Melo, Moacir Fernandes Queiroz, Lucas Alighieri Neves Costa Batista, Pablo Castro Santos, Mariana Santana Santos Pereira Costa, Jailma Almeida-Lima, Rafael Barros Gomes Camara, Leandro Silva Costa, Hugo Alexandre Oliveira Rocha

**Affiliations:** 1 Laboratory of Natural Polymer Biotechnology (BIOPOL), Department of Biochemistry, Center of Biosciences, Federal University of Rio Grande do Norte (UFRN), Natal, Rio Grande do Norte- RN 59078-970, Brazil; dayanne.oliveira@ifpi.edu.br (D.L.G.); melo.krt@gmail.com (K.R.T.M.); moacirfqn@gmail.com (M.F.Q.); lucasalighieri@gmail.com (L.A.N.C.B.); biolottus23@yahoo.com.br (J.A-L.); rafael_bgc@yahoo.com.br (R.B.G.C.); 2 Federal Institute of Education, Science and Technology of Piauí (IFPI), São Raimundo Nonato Campus, São Raimundo Nonato-PI 64.770-000, Brazil; 3 State University of Rio Grande do Norte (UERN), Mossoró-RN 59.610-210, Brazil; pablodecastrosantos@gmail.com; 4 Federal Institute of Education, Science and Technology of Rio Grande do Norte (IFRN), João Câmara-RN 59.550-000, Brazil; mariana.costa@ifrn.edu.br; 5 Federal Institute of Education, Science and Technology of Rio Grande do Norte (IFRN), Canguaretama-RN 59.190-000, Brazil; leandro.costa@ifrn.edu.br

**Keywords:** urolithiasis, green algae, COD dumbbell, COD stabilization

## Abstract

Urolithiasis affects approximately 10% of the world population and is strongly associated with calcium oxalate (CaOx) crystals. Currently, there is no efficient compound that can be used to prevent this disease. However, seaweeds’ sulfated polysaccharides (SPs) can change the CaOx crystals surface’s charge and thus modify the crystallization dynamics, due to the interaction of the negative charges of these polymers with the crystal surface during their synthesis. We observed that the SPs of *Caulerpa cupressoides* modified the morphology, size and surface charge of CaOx crystals. Thus, these crystals became similar to those found in healthy persons. In the presence of SPs, dihydrate CaOx crystals showed rounded or dumbbell morphology. Infrared analysis, fluorescence microscopy, flow cytometry (FITC-conjugated SPs) and atomic composition analysis (EDS) allowed us to propose the mode of action between the Caulerpa’s SPs and the CaOx crystals. This study is the first step in understanding the interactions between SPs, which are promising molecules for the treatment of urolithiasis, and CaOx crystals, which are the main cause of kidney stones.

## 1. Introduction

Urinary lithiasis or urolithiasis is a pathophysiological condition resulting from the formation of kidney stones. The incidence of urinary lithiasis in the world is increasing rapidly. In the last decade, prevalence doubled from approximately 6% to 11% among men and 4% to 7% among women [1]. One of the major problems with urolithiasis is the risk of developing chronic kidney disease (CKD) and end-stage renal disease (ESRD), a serious form of CKD [2]. This disease has a high social and economic cost, since, in the USA alone, CKD treatment exceeds $50 billion a year [3]. In England, according to a recent report published by the National Health Service (NHS) Kidney Care, costs with CKD case management exceeds those of breast, lung, colon and skin cancers combined [4].

Urolithiasis can be influenced directly by the types of formed crystals, as each crystal has its own different ability to bind to the renal epithelium and to initiate the formation of the stones. Calcium oxalate (CaOx) crystals make up about 70% of urinary stones [5], and they are found in three different forms: monohydrate (COM), dihydrate (COD) and trihydrate (COT). The COM crystal is thermodynamically stable, exhibiting monoclinic geometric morphology (which, in optical microscopy, can be visualized with a rectangular shape) and showing greater affinity with the renal epithelium. For these reasons, they are found in kidney stones at a frequency twice as high as that of COD crystals [6,7]. The COD are commonly found in the urine of asymptomatic persons for urolithiasis and present a characteristic tetrahedral bipyramidal morphology, which can be easily visualized by light microscopy [7]. COT crystals show drusiform morphology. They are unstable, and therefore are rarely found in the urine of patients [8,9].

**Figure 1:**
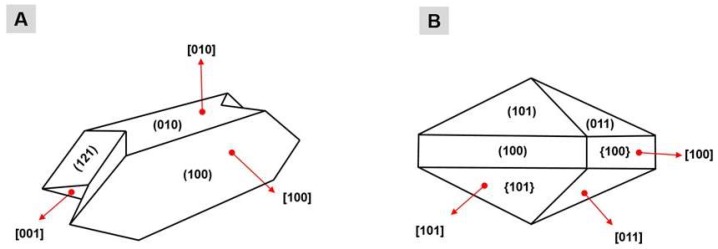
Representation of the faces of the calcium oxalate crystals: (**A**) crystals COM; and (**B**) crystals COD. The numbers presented between parentheses represent each face of a crystal; the numbers displayed between brackets represent the direction of growth of each face of a crystal; and the numbers presented between braces represent the set of same faces of a same crystal.

The formation of these CaOx crystals is derived from a physicochemical process divided into three phases: nucleation, aggregation and crystal growth. The preponderant condition for crystal formation (nucleation) is urinary supersaturation due the superfluous ions that cannot be stored in the buffer system; the condensation of these salts occurs forming tiny crystals, which are called nuclei. The aggregation occurs by the union of one or more growing nuclei, which form crystals of larger dimension and mass that can precipitate in the renal epithelium, being able to adhere to or internalized by cells [9]. The growth of the kidney stone occurs by aggregation of preformed crystals or by secondary nucleation of a crystal adhered to the intrarenal structures [10]. The thickness of a crystal is related to the proportion of its faces. The CaOx crystals have three growth faces, and each face differs in size and are named according to the type of crystal: faces (100), (010) and (121) for the COM crystals (Figure 1A); and faces (100), (101) and (011) for the COD crystals (Figure 1B).

The changes in the CaOx crystal’s face are caused by the other molecules present in the urinary tract, considering the fact that the concentration of ions in healthy persons and lithogenic patients is practically the same. These molecules, such as citrate, pyrophosphate, glycosaminoglycans and glycoproteins, present in the renal and urinary ducts, have the function of stabilizing CaOx crystals, mainly in the COD morphology [11,12].

Recently, seaweeds sulfated polysaccharides (SPs) have begun to be evaluated for their anti-lithic capacity. The studies are done in vitro as well as with cells under culture conditions and in vivo. The data are well reviewed in a recently published paper [13], which verifies these polymers can inhibit the formation of CaOx renal stone formation in different ways: they inhibit crystallization (both in the nucleation phase [14,15,16] and in growth phase [16,17]); they inhibit the aggregation phase [14,15,16,17,18]; they inhibit the occurrence of COM crystals and the transformation of COM to COD [14,16,17,18]; and they inhibit renal tubular cell injury in vivo [19,20,21]. 

The Brazilian coast has a wide diversity of marine seaweed, especially on the northeast coast; however, the SPs of few species are characterized and evaluated for their therapeutic potential. The SPs (CCB-0.3, CCB-0.5, CCB-1.0 and CCB2.0) from *Caulerpa cupressoides* var. *flabellata* have been studied by our research group for almost a decade. 

Initially, we showed that SPs-rich extract from *C. cupressoides* exhibited several biological activities, including anticoagulant, antiproliferative and antioxidant activities [22]. Later, four distinct SPs populations, named CCB-0.3, CCB-0.5, CCB-1.0 and CCB2.0 were obtained from this SPs-rich extract. These SPs showed strong immunostimulatory activity [23]. In addition, the SPs CCB-F0.3 and CCB-F0.5 exhibited antioxidant activity and significant iron chelating ability [24]. However, the effect of these SPs on the formation of CaOx crystals has not yet been described. It is noteworthy that, thus far, no SP of green seaweed has had its action on crystal formation evaluated. Based on these considerations, the objectives of the present study were to obtain SPs from *C. cupressoides* (CCB-F0.3, CCB-F0.5, CCB-F1.0 and CCB-F2.0), evaluate their effect on crystallization of CaOx in vitro, and elucidate the different morphological characteristics of the formed CaOx crystals to propose a model of interaction between the populations of SPs obtained with the CaOx crystals.

## 2. Results and Discussion

### 2.1. C. cupressoides’s Sulfated Polysaccharides Extraction, Chemical and Physicochemical Analysis 

**Figure 2:**
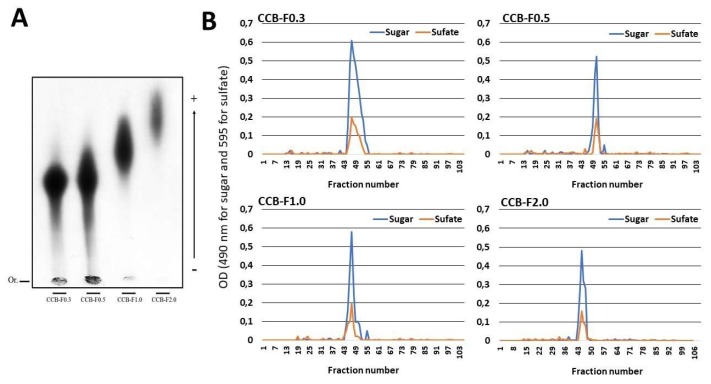
(**A**) Electrophoretic mobility of the sulfated polysaccharides from *C. cupressoides* seaweed. Fifty micrograms of the Caulerpa SPs were applied on agarose gel prepared with 1,3-diaminopropane-acetate buffer 0.05 M, pH 9.0, and then subjected to electrophoresis at 90 V/cm for 60 min. The gel was maintained in 0.1% cetyltrimethylammonium bromide for 2 h, dried and subsequently stained with 0.1% toluidine blue (in 50% ethanol and 1% acetic acid in water) for 15 min. The gel was unstained with the same staining solution without the dye. (**B**) Gel permeation chromatography of sulfated polysaccharides from *C. cupressoides*. Two hundred micrograms of the Caulerpa SPs were applied to a Sephadex^®^ G-100 column (140 cm × 1 cm). The column was eluted with 0.2 M acetic acid, 1 mL fractions were collected, and the presence of sugars and sulfate was determinate as described in Methods Section.

In a previous study [23,24], our group extracted four populations of sulfated polysaccharides from the *C. cupressoides* seaweed. These populations were named as CCB-F0.3, CCB-F0.5, CCB-F1.0, and CCB-F2.0. In this work, using the same previously described methodology, we also obtained four polysaccharide populations from *C. cupressoides*. These samples were subjected to agarose gel electrophoresis in PDA buffer. Figure 2A shows an electrophoresis slide stained with toluidine blue. We can see the four populations stained with toluidine blue, indicating that they are constituted of SPs. Different electrophoretic mobility can also be verified: CCB-F0.3 and CCB-F0.5 are polysaccharides of low mobility in comparison with the others, CCB-F1.0 has intermediate mobility, and CCB-F2.0 is the population with higher mobility. By comparing our data with the electrophoresis slide presented by Costa and colleague [24], we can confirm the identity of the polysaccharides obtained.

The Caulerpa SPs were further analyzed by gel permeation chromatography (GPC) in a Sephadex^®^ G-100 column to determine their homogeneity (Figure 2B). The chromatograms of the Caulerpa SPs showed a single peak. Furthermore, the chromatogram obtained from GPC was used to calculate the apparent molecular weight using a regression equation determined using different molecular weight standards. Thus, the molecular weight of CCB-F0.3; CCB-F0.5; CCB-F1.0; and CCB-F2.0 was found to be 150, 130, 155, and 165 kDa, respectively. These values were similar those demonstrated by Costa and colleague [24]. However, they observed that these polysaccharides had polydispersions greater than the ones observed here. 

As observed previously [24], in all extracted SPs, the protein contamination was below 0.1% (Table 1), confirming the efficacy and reproducibility of the extraction methodology. 

Table 1 reveals a higher sulfate/sugar ratio for CCB-F0.3 (~1.10) and the lowest ratio for CCB-F2.0 (~0.72). These values differ from those previously observed [24], where the authors found CCB-F0.5 with higher sulfate/sugar ratio and CCB-F1.0 with the lowest ratio of the four fractions. These data were not surprising, since many authors report changes in the chemical compositions of polysaccharides extracted from the same species of seaweed when collected at different sites [25,26]. However, the seaweed collected by Costa and colleagues [24] as well as here was obtained at the same site (6°1’8.19”S–35°6’33.40”W), which discards this possibility, even though they were collected in different years, thus the seaweed was exposed to different environmental conditions, leading to changes in the SPs composition. This effect has already been reported by different authors, but these variations are not homogeneous; each species of seaweed responds differently to the environmental changes in the collection site. For example, the green seaweed *Ulva fasciata’s* SPs varied in yield, monosaccharide composition and sulfate amount [27], while *Delesseria sanguínea*’s SPs’ monosaccharide composition and sulfate content have not been affected by seasonality [28].

We believe that the factor responsible for this difference in our data compared to those previously published [24] can be explained by the polydispersity of polysaccharides seen by CPC. The polysaccharides studied by Costa et al. [24] are more polydispersed than the ones studied here because they have oligosaccharides in their constitution. These oligosaccharides present different monosaccharide composition, and, therefore, the data of the chemical analysis of the polysaccharides shown by Costa et al. [24] were different from those presented here.

### 2.2. Effect of Sulfated Polysaccharides C. cupressoides on the Formation of Calcium Oxalate Crystals

We induced the formation of CaOx crystals in the presence of SPs of *C. cupressoides* to evaluate the effect of SPs on the formation of these crystals. Based on light field optical microscopy, we could infer the effect of SPs on the formation of crystals by quantification of each type of crystal. The data obtained are summarized in Table 2.

When we analyzed the data shown in the table, we found that the presence of polysaccharides increased the amount of crystals formed. In the presence of CCB-F0.3, the amount of crystals increased 12-fold, followed by CCB-F1.0, which increased by approximately 9-fold. However, despite the increase in the amount of crystals, the size of the crystals formed when treated with *C. cupressoides* seaweed SPs was reduced about four-fold on average, reaching, in some cases, about 1 µm after treatment (Table 2). The CCB-F0.3 again stood out by decreasing seven times the size of COM and five times the size of COD.

Not every anionic molecule can interact with the oxalate crystals and interfering with their formation [29]. However, when they interact with the crystals, changes in the morphology [14,15] and size of the crystals can occur [16,17,29]. For example, Wesson and collaborators [29] demonstrated that anionic proteins tend to increase the number of crystals and decrease their size since these negatively charged proteins interact more with the rich calcium faces of both COM and COD crystals, blocking their growth. In addition, the anionic molecules induce repulsions between the formed nuclei (the nano/micro crystals), preventing the aggregation process, as they are associated with the crystals. 

With respect to the large number of crystals formed in the presence of *C. cupressoides* SPs, we believe that as the ions are not completely consumed during nuclei formation, these are available for the formation of new nuclei. These factors together justify the observation of the large number of small crystals formed in the presence of *C. cupressoides* SPs.

In addition, in Table 2, we can see that all obtained SPs (except CCB-F2.0) induced the formation of an amount of COD-type larger than COM-type, and, again, CCB-F0.3 was highlighted, as in the presence of CCB-F0.3, the number of crystals was 209 ± 12.3 units of COD type, much higher than the number of COM type (48 ± 10.9 units), making a ratio of approximately four COD for each COM.

It is important to emphasize that COM-type crystals, when formed in vivo, can be concentrated in the renal tubular fluid and interact with kidney tubular epithelial cells, giving them enough time to grow on the cell surface or to aggregate mutually to form large crystals, which finally leads to the formation of kidney stones. On the other hand, COD crystals feature a minimal contact area with the renal epithelium and, therefore, bind the cell membrane in a smaller amount [30].

Other SPs of different seaweeds showed the ability to inhibit the formation of COM crystals or to stabilize the dihydrate form of the CaOx crystal (COD). For example, Ouyang and colleagues [14] worked with the edible seaweed *Laminaria japonica* and found that its native and modified SPs induced the formation of only COD crystals [14]. However, SPs from *Dictiopteris justii* can inhibit the formation of CaOx crystals. In addition, its sulfated glucan was also able to stabilize CaOx crystals in the COD form [16]. There is no hypothesis that explains how SPs stabilize CODs. However, studies with carboxylated polymers with the same net charge showed that they do not influence the COD stability in the same way, and it was proposed that the distribution of the charges around the molecule was a more important factor than the charge alone during this process of stabilization [31]. Thus, this should probably be an important factor for SPs as well.

### 2.3. Morphology of the Crystals Formed in the Presence of Caulerpa polysaccharides

**Figure 3:**
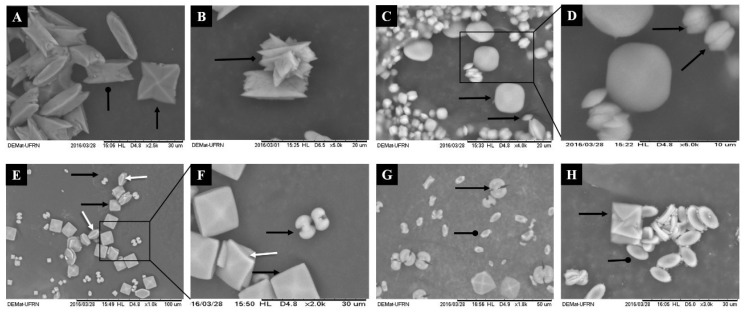
Scanned Electron micrographs of the crystals formed morphotypes after incubation with polysaccharides of green seaweed *C. cupressoides*. The CaOx crystals were formed in a stable CaOx solution target (1 mM) in the absence (**A**,**B**) and in the presence of SPs *C. cupressoides* (0.25 mg/mL): (**C**,**D**) CCB-F0.3; (**E**,**F**) CCB-F0.5; and (**G**) CCB-F1.0; (**H**) CCB-F2.0. Black arrow shows the COD form; larp-arrow rounded head shows the COM shape; arrow tipped diamond shows the COT form. White arrow indicates tetragonal bipyramid structures with thicker (100) faces.

For a more detailed analysis of the morphological characteristics of the formed crystals, the images were obtained by scanning electron microscopy (SEM) and were analyzed subsequently. The results are summarized in Figure 3.

The images in Figure 3 corroborate the results of Table 2, showing that there was an increase in the amount of formed crystals, which were also smaller in size. In agreement with Table 2, there were more COD-type crystals in the crystal images formed after incubation with CCB-F0.3, CCB-F0.5 and CCB-F1.0 (Figure 3C–G) when compared to control crystals (Figure 3A,B). The crystals that were formed without the incubation with the SPs (control) had the morphological characteristic of COM and COD, marked by isotropic growth on all the faces of the crystals. Moreover, we observed control only in the crystals of the COT-type (arrow tipped diamond, Figure 3B), i.e., all treatments with seaweed *C. cupressoides* SPs inhibited the formation of this crystal.

Regarding the COD crystals, after treatment, they showed a rounder shape (without well-defined tips) than the control group. CCB-F0.3 (Figure 3C) induced the formation of nearly round, spherical crystals with the tiny (100) faces, evidenced by the arrow in Figure 3D. The CCB-F2.0 and CCB-F1.0 polysaccharides induced the formation of COD, which assumed the tetragonal bipyramid shape, but with the tips slightly rounded. CCB-F0.5 induced the formation of crystals with tetragonal bipyramid structures with thicker (100) faces (white arrow with arrowhead, Figure 3E,F); besides, these crystals had rounded edges when compared to COD formed in the control.

Regarding COD crystals, we also observed that both CCB-F0.5 and CCB-F1.0 induced the formation of a mixture of COD crystals, which, in addition to the already described types, also formed dumbbell-shaped crystals. These crystals showed two hemispheres connected with a rod (Figure 3F). COD crystals with traditional bipyramidal structure have the face (101) as the dominant face. According to Thomas and colleagues [32], the COD crystals only take the form of dumbbells when the ratio between faces {100} and {101} is greater than 1, that is, the face (100) grows much more than the face (101). These authors also demonstrated that the presence of polyacrylate (PAA) also promotes the formation of COD crystals in the form of dumbbells. This indicates that COD crystals in the form of dumbbells can be formed in the presence of some anionic compounds. However, we still do not know what SP structural features are required for this to occur.

Regarding COM crystals, when formed after treatment with *C. cupressoides* SPs, they were smaller and had structure with the faces {100} dominating. There was an increase in growth in the direction [010] rather than growth in the direction [100]. This gave COM edges and tips with less sharp angles, as they were elliptical (oval in the form of a plaque). Thus, the morphological characteristic assumed by COM crystals after incubation with *C. cupressoides* SPs is advantageous, as, by leaving the edges of their faces with a slight angle (rounded), the possible interaction of these crystals in renal epithelium could be decreased, which would consequently favor their passage in the urinary tract, thus reducing the formation of kidney stones. In agreement with our hypothesis, studies analyzing CaOx crystals in the urine of lithogenic patients verified that the COM crystals had edges and tips with sharp angles and concluded that these are important factors for the anchorage of these crystals in the renal epithelium [30,33].

### 2.4. FT-IR Spectrum Analyses

**Figure 4:**
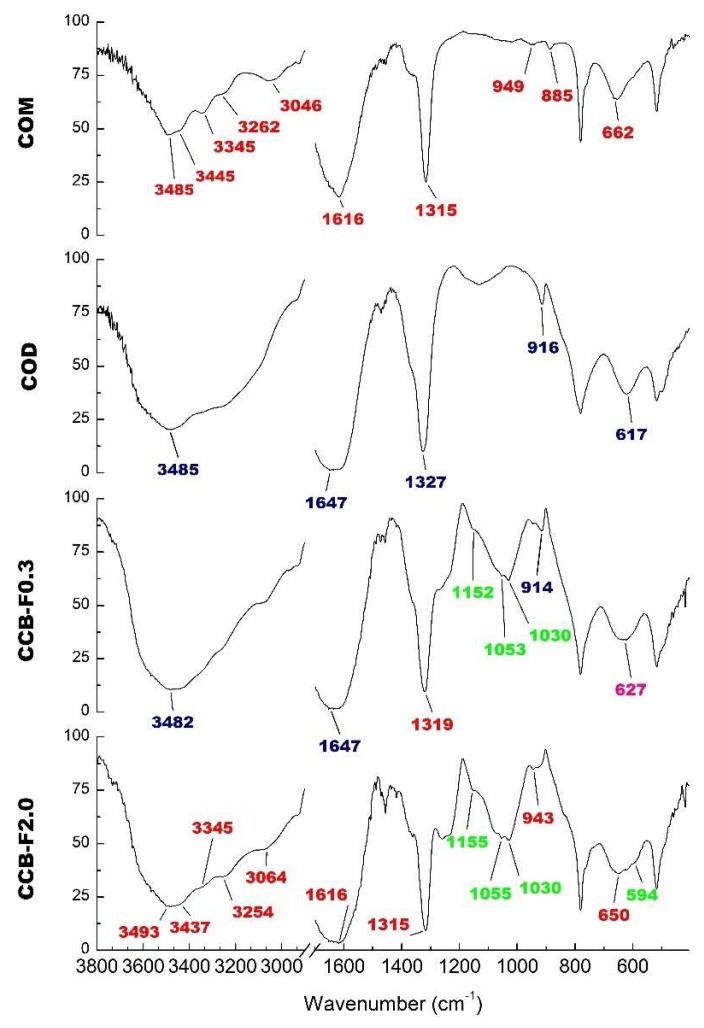
Infrared spectrum of COM crystals, COD crystals and CaOx crystals formed after incubation with *Caulerpa* SPs (CCB-F0.3 and CCB-F2.0). The values in red indicate the bands that are characteristic of the COM crystals; those in blue are characteristic of COD crystals; in green are the values that indicate the presence of sulfate; and in pink is the band found in the spectra of both crystals (COM and COD).

The COM and COD crystal FT-IR spectra are shown in Figure 4. We observed that the main difference between these spectra was the region between 3040 and 3500 cm^−1^. While, in COM crystal spectra, we observed at least five prominent bands (Figure 4) in this same region, in COD crystal spectra, we only observed one great intensity band around 3485 cm^−1^ (Figure 4, COD spectra). Other typical signals of COM crystal were observed around 947, 885 and 663 cm^−1^ (Figure 4, COM spectra), whereas characteristic COD signals were observed at 916 and 609 cm^−1^. Both spectra are similar to those previously described for COM and COD crystals [34,35].

Since we observed that more COD crystals were formed in the presence of CCB-F0.3 and more COM crystals were formed in the presence of CCB-F2.0, these samples were chosen to perform infrared analysis. In the spectra of these crystals (Figure 4, CCB-F0.3 and CCB-F2.0 spectra), the values in red indicate the bands that are characteristic of the COM crystals; those in blue are characteristic of COD crystals; in green are the values that indicate the presence of sulfate; and in pink, we show the band that we found in the spectra of both crystals (COM and COD). In this way, there are some bands in both spectra that we also observed in COM spectra (red values) or COD spectra (blue values). However, these bands have slightly different values. This was probably due to the presence of polysaccharides. In addition, there are bands (green values) around 1232–1256 cm^−1^ that indicate asymmetric vibration S=O [24,36], and the bands around 1150 and 1025–1033 cm^−1^ are indicative of vibration associated to C–O–S–O [24,37]. These bands were also observed in the spectra of the crystals obtained in the presence of the other SPs (data not shown) and confirmed the presence of SPs CCB-F0.3, CCB-F0.5, CCB-F1.0 and CCB-F2.0 in CaOx crystals.

Spectra analysis also allowed noticing that, in the presence of CCB-F0.3, there was a predominant formation of COD crystals, since a unique band was observed in the region between 3040 and 3500 cm^−1^. However, in the CCB-F2.0 spectra, it was possible to observe multiple bands in this region, indicating that SPs were mainly complexed with COM crystals. Thus, these data are in line with those obtained by microscopic analysis.

### 2.5. Zeta Potential

The zeta potential (ζ) of crystals formed in the presence of *C. cupressoides’* SPs was measured to verify if changes in the number and/or morphology of crystals were associated with changes in the surface charge of these crystals. The results are shown in Table 3.

The ζ mean value of untreated CaOx crystals was +8.85 ± 3.30 mV. This positive profile of crystal charge surface can be mainly related with the presence of calcium ions in the crystal structure. All SPs decreased the zeta potential of CaOx crystals, ranging from −25.82 ± 6.36 mV in the presence of CCB-F0.3 to −68.70 ± 12.01 mV in the presence of CCB-F2.0.

An interesting fact is that the increase of crystals ζ did not correspond to the sulfate/sugar ratio (Table 1), that is, polysaccharide with high amounts of sulfate groups did not promote formation of crystals with lower ζ value. Other authors have already noted this fact [15,16], and it is proposed that SP associated with crystal tends to assume a conformation that allows it to have higher or lower exposition of its charged groups. Therefore, it is a situation where, in a negatively charged polysaccharide, it may assume a conformation in which its charges are not so exposed on the crystal surface, resulting in ζ of the crystal being closer to zero.

The ζ increased with the presence of SPs of *C. cupressoides*, which explains part of the formation of many small crystals. The elevated negative charge on the crystal surface led to repulsion of other crystals and blocked crystal aggregation and growth. This interference in crystal aggregation/growth has also been observed by other authors while working with other seaweeds SPs [15,16]. In those works, the formation of many small crystals and elevated crystal negative charge are also reported.

Zeta potential analysis provides great indications that SPs interact with CaOx crystals structure. However, there are no studies that prove direct binding between SPs from seaweed and CaOx crystals. However, this connection is well described for other molecules that also have negative charges in their structure, such as polyacrylate [32], osteopontin [38,39], glycosaminoglycans [40], and citrate [41]. Therefore, it can be confirmed, based on the data presented here and compared with those in the literature, that SPs seaweeds are able to alter the surface charge of CaOx crystals and thereby modify their crystallization dynamics.

### 2.6. Fluorescence and Flow Cytometry Analyses of CaOx Crystals and FITC-Labeled SPs

In another set of experiments, the SPs were covalently conjugated to the FITC, as described in Section 3.8, and used as fluorescent probes with the goal of observing the binding of SPs to the formed crystals. To this end, FITC-labeled polysaccharides were incubated with the supersaturated solutions inducing the formation of CaOx crystals, and the crystals resulting from this incubation were analyzed by flow cytometry and fluorescence microscopy. Crystals formed in the presence of fluorescently unlabeled SPs (0.1 mg/mL) and FITC (0.1 mg/mL) were used as control.

**Figure 5:**
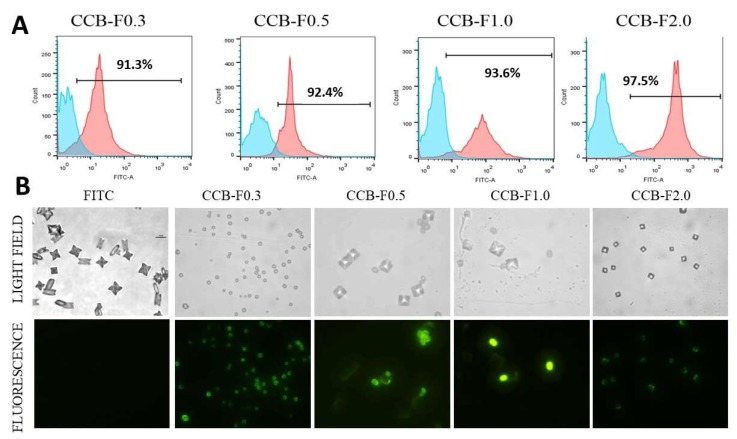
Fluorescent crystal analyzes by flow cytometry and fluorescence microscopy. (**A**) CaOx crystals were formed in the presence of fluorescently unlabeled SPs (0.1 mg/mL) and FITC (0.1 mg/mL) (blue graphics) or in the presence of fluorescent SPs (0.1 mg/mL) (pink graphics) and detected by flow cytometry. The number within each square corresponds to the percentage of crystals labeled fluorescently. (**B**) Comparison between crystals in light field microscopy (non-fluorescent), observed in the upper line, and the SPs-FITC-conjugated crystals (fluorescent in green), observed in the bottom line. FITC corresponds to CaOx crystals formed in the presence of FITC (0.1 mg/mL). These crystals were not fluorescent. The assays were performed twice (*n* = 3).

As expected, crystals formed in the presence of inflorescent SPs and FITC were not detected by flow cytometry, indicating that FITC alone did not bind to CaOx crystal. However, using this technique, we verified that, in all condition evaluated, more than 90% of crystals formed in the presence of fluorescent SPs showed positive FITC signal (Figure 5A).

When those crystals were analyzed by fluorescence microscopy, we observed that FITC alone did not label the crystals and did not change the crystal morphology. On the other hand, we noticed that the crystals appeared marked by FITC (green) in almost totality (Figure 5B) when formed in the presence of FITC-labeled polysaccharides; besides, we were able to differentiate formed COM and COD crystals. These data give evidence that SPs interacted with the entire crystalline network, probably with the calcium ions that were present in these crystals.

### 2.7. Stabilization of COD Crystals

The data presented thus far show that, except for CCB-F2.0, all other SPs of *C. cupressoides* induced higher amount of COD crystals in comparison to the positive control. In addition, SPs gave COD crystals’ higher stability. This behavior was already observed by Escobar and colleagues [42], in the presence of other sulfated (dermatan sulfate, oversulfated heparin and keratan sulfate) and phosphorylated (phosphorylated chitosan) polysaccharides. Interestingly, there was a greater formation of COM crystals when these authors used desulfated dermatan, chitosan and hyaluronic acid (carboxylated, but not sulfated polysaccharide). These results show the importance of sulfate groups in polysaccharides for the stabilization of COD crystals [42].

However, not every sulfated polysaccharide promotes stabilization of COD crystals, as shown by Melo and colleagues [16] and by our results, since CCB-2.0 induced more COM formation. This showed that the SPs’ COD stabilizing effect was not merely a charge effect, but also depended on how charges were distributed across the polysaccharide chain. Moreover, these data show that COD formed in the presence of the different *C. cupressoides* SPs may take different forms, which seems to indicate that SPs can stabilize COD in different ways, perhaps because they associate with crystals in different faces.

**Figure 6:**
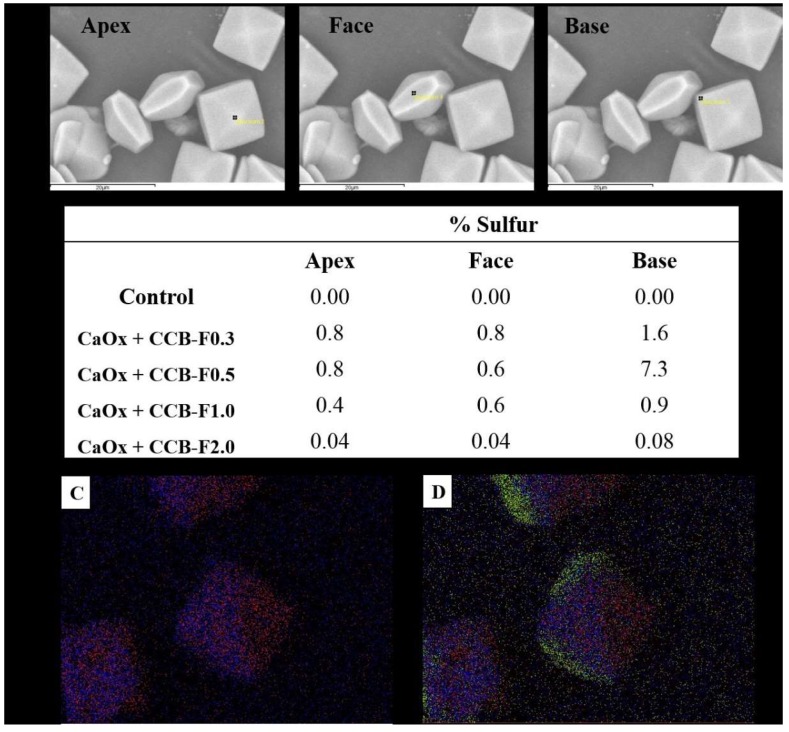
Crystal surface atomic composition characterization by EDS. (**A**) SEM image of CaOx with the presence of CCB-F0.5. The black squares (apex, face, and base) indicated the point where the EDS analyses were performed to quantify the sulfur atoms. (**B**) Percentage of sulfur distribution in different parts of CaOx and CaOx treated with polysaccharide. (**C**) Calcium (red dots) and oxygen (blue dots) marking in CaOx with the presence of CCB-F0.5. (**D**) Increase in sulfur marking (green dots) in CaOx with the presence of CCB-F0.5.

To confirm this hypothesis, the surfaces of the COD crystals obtained after the incubation with the SPs studied here had their atomic composition characterized through the spectroscopic chemical microanalysis of X-rays by energy dispersion (EDS). The sulfur atoms were quantified at different points of the crystals: apex, face and base (Figure 6A). The sulfur found on the surface of the crystals should represent the sulfate (SO_4_^2-^) groups of SPs, since CaOx is composed only of calcium, carbon and oxygen, thus some considerations were made.

The results from this analysis are summarized in the table of Figure 6B. We observed that three situations occurred in the sulfur distribution: the first occurred in the crystals formed with CCB-F0.3 and CCB-F2.0, where there was twice the amount of sulfur in the region of the base than in the other portions; the second occurred with the crystals formed with CCB-F0.5, where there was almost ten times more sulfur at the base of these crystals than at the apex or face; and the last situation was when there was a different distribution at the base, face and apex, with the amount of sulfur at the base being twice as large as the apex, as was the case with CCB-F1.0.

We found no other articles that report this type of analysis; therefore, it was not possible to compare our results with those of other authors. However, some considerations were made.

Is it necessary for the SPs to be distributed differently throughout the crystal so that there is a greater stabilization of the COD crystals? It does not seem so, since CCB-F1.0 had this type of distribution, but the COD:COM ratio obtained with this sample was only 2:1 (Table 2).

The other SPs were more concentrated on the base. However, there is a subtle relationship between the amount of sulfur that should be at the base, apex and face. If there was a lot of sulfur in the base (about ten times more) than in the other points, as occurred with the presence of CCB-F0.5, there was stabilization of COD, but with only a COD:COM ratio of 3:1 (Table 2). The ideal distribution for COD stabilization seemed to be that observed with CCB-F0.3, since the COD:COM ratio was 5:1. The CCB-F0.3 concentrated more on the base, but its amount was only twice of that observed in the other points; in addition, the amount of this SP at the apex and face was similar. This distribution profile was also observed with CCB-F2.0. However, we found that the crystals formed with this polysaccharide had twenty times less sulfur than the crystals formed with CCB-F0.3.

## 3. Materials and Methods 

### 3.1. Sulfated Polysaccharides Extraction from Green Seaweed C. cupressoides

The seaweed, *Caulerpa cupressoides* var. *flabellata*, was collected from Nísia Floresta, on the southern coast of the state of Rio Grande do Norte, Brazil (S 6°1′819′′ and W 35°6′33.40′′) and then transported to the Laboratory of Natural Polymer Biotechnology, Department of Biochemistry, Federal University of Rio Grande do Norte, UFRN. The seaweed was identified according to its morphology [43]. The material collection occurred under authorization of Brazilian National Management System Genetic Heritage and Associated Traditional Knowledge (loose translation) SISGEN n° A0D4240.

After collection, the green seaweed *C. cupressoides* var. *flabellata* Børgesen was cleaned with running water and oven dehydrated at 45 °C. It was sprayed and then treated four times with two volumes of ethanol for depigmentation and delipidation of the material. Two volumes of 0.25 M NaCl were added to the powder obtained, with the pH being adjusted to 8.0. The proteolytic enzyme maxatase (60 °C, for 18 h) was added to this material. The suspension was then centrifuged at 10,000× *g* for 20 min. The precipitate was discarded, and the volume of the supernatant was measured fractionated with increasing volumes of acetone, obtaining SPs according to a method established by Costa and collaborators [24].

### 3.2. Chemical and Physicochemical Analysis

The total sugars were determined according to Dubois and collaborators (1956) by the phenol–H_2_SO_4_ reaction, as described previously [24]. The sulfate content was determined based on the gelatin/barium method [44]. Protein quantification was determined using Spector’s method [45]. 

### 3.3. Agarose Gel Electrophoresis in 1,3-Diamino Propane Acetate Buffer (PDA)

The electrophoretic mobility of *C. cupressoides* SPs was evaluated by gel electrophoresis in PDA buffer, according to the reported method [46]. Initially, glass slides were coated with 0.6% (*m*/*v*) agarose in PDA buffer (0.05 M, pH 9.0). Subsequently, aliquots of the polysaccharides (about 50 μg) were applied to the gel and subjected to electrophoresis (100 V, 4 °C) for 60 min. After the electrophoretic run, the polysaccharides were precipitated with 0.1% cetyltrimethylammonium bromide (CETAVLON, Sigma Chemical Company, St. Louis, MO, USA) for 2 h at room temperature and the gels were dried using warm air stream. To visualize the SPs, gels were stained with a solution of 0.1% toluidine blue in 1% acetic acid and 50% ethanol. The gel was then de-stained with the same solution without the dye. Three independent analyzes were performed.

### 3.4. Gel Permeation Chromatography (GPC)

The molecular weight and homogeneity of samples were determined by gel permeation chromatography (GPC). Each sample was dissolved to a final concentration of 10 mg/mL and 200 µg were applied to a column containing Sephadex G-100 (130 × 1 cm i.d., Sigma Chemical Company, St. Louis, MO, USA). GPC was performed using an isocratic elution mode. The molecular weight was estimated by reference to a calibration curve made by dextran sulfate standards (10, 40, 70, 147 and 500 kDa) (Sigma Chemical Company, St. Louis, MO, USA). Homogeneity of samples was evaluated by chromatographic profile. The total sugars were determined according to Dubois and collaborators (1956) by the phenol–H2SO4 reaction, and the sulfate was determined by metachromasia, as described previously [24].

### 3.5. Fourier Transformed Infrared (FT-IR) Spectroscopy Analysis

The infrared spectra of the CaOx crystals controls formed after incubation with the SPs of *C. cupressoides* were obtained using infrared spectroscopy via Fourier transform (IRAffinity-1 spectrometer, Shimadzu Corp., Kyoto, Japan) equipped with the IRsolution 1.20 software. SPs and crystals samples (5 mg) were completely mixed with dried potassium bromide powder (KBr) and then compressed. The sweep frequency range was 4000–400 cm^−1^. Thirty-two scans at a resolution of 4 cm^−1^ were evaluated and referenced against air. The Infrared Spectroscopy Analysis was carried out at the Department of Chemistry of the Federal University of Rio Grande do Norte.

### 3.6. Calcium Oxalate Crystallization Assay

The CaOx crystals can be formed in vitro from Ca^2+^ and oxalate to a mixture of calcium chloride (8 mM/L), sodium oxalate, sodium chloride (200 mM/L) and sodium acetate (10 mM/L) as final concentrations of this solution from physiological concentrations of urine. The formation of the CaOx crystals was evaluated in the presence or absence (control) of *Caulerpa*’s polysaccharides (0.1 mg/mL) [15]. When necessary, crystal formation was also induced in the presence of 0.1 mg/mL fluorescein isothiocyanate (FITC) or in the presence of SPs and FITC. These crystals were centrifuged at 5000× *g* and the supernatant was discarded. The precipitate was composed mainly of crystals of CaOx, and was resuspended in 0.5 mL of water, and a solution of 0.1 mL was placed on a histological slide and observed under an optical microscope (600×). Images of 10 different fields were obtained for each slide, and then the crystal diameters and sizes were analyzed using the NIS Elements AR Ver4.30.01 DU1 64-bit software, 2014 (Melville, NY, USA). The conclusions about the measurements of the CaOx analyzes were obtained after a trial of distinct experiments, being repeated four times.

### 3.7. Scanning Electron Microscopy (SEM) and Dispersive Energy Spectroscopy (EDS)

To observe the superstructure and composition of generated crystals in the presence of the SPs of *C. cupressoides*, scanning electron microscopy (Hitachi Tabletop Microscope TM-3000 model, with 5 kV voltage acceleration, 50/60 Hz of frequency, image magnification 15–30,000, Hitachi Chemical Company, Ltd., Tokyo, Japan) and dispersive energy spectroscopy (Swift ED TM-3000, Oxford Instruments, Abingdon, Oxfordshire, UK) images were generated with 1280 × 960 pixels resolution. The scanning electron microscopy was carried out at the Department of Materials Engineering of the Federal University of Rio Grande do Norte. The images showed in the results are representative of three independent experiments.

### 3.8. Zeta Potential (ζ) Measurements

After 30 min of crystal formation in the presence and absence of the polysaccharides [16], the solutions were centrifuged (5000× *g*). The crystal concentrate was then suspended in 1.5 mL of ultrapure water (pH ~7.0), and the zeta potential of the ζ samples was obtained using a Zeta Plus^®^ analyzer (active temperature control: −5 °C at 110 °C, ±0.2 °C, 1 at 4 s/cycle; pH range: 2 at 12; conductivity: 0 at 7.5 mS/cm; and intensity of the electric field: 0 at 3.2 kV/m) (Brookhaven instruments, Holtsville, NY, USA). Since pH value and ionic strength are factors that affect zeta potential results, the values of these two parameters, in all solutions evaluated, were always the same.

### 3.9. Light Microscopy and Fluorescence Microscopy for the Analysis of CaOx Crystal Morphology

To better understand the morphology and arrangement of the SPs in the CaOx crystals, polysaccharide was labeled with fluorescein isothiocyanate (FITC). Five milligrams of each polysaccharide were added to 0.1 mL of phosphate buffer (PBS) at pH 7.0 containing 0.1 mg FITC. The solution was kept in an environment with reduced brightness, at room temperature for 1 h. The material was then labeled with deionized water in membranes with pores of 6 kDa, and then lyophilized. Samples without SPs as well as samples of FITC-labeled polysaccharides were not subjected to new production of CaOx crystals and slides, assembled according to Section 3.6. Images were captured from different fields under a fluorescent microscope (TE-Eclipse 300, Nikon, Melville, NY, USA). We performed three different experiments.

### 3.10. Statistical Analysis 

All data are presented as the mean ± standard deviation (*n* = 3). The ANOVA test was performed to check the difference between results. The Student–Newman–Keuls test (*p* < 0.05) was used to solve similarities found by ANOVA. All tests were performed in GraphPad Prism 5 (GraphPad Softwares, San Diego, CA, USA).

## 4. Conclusions

Four sulfated polysaccharide populations were extracted from the seaweed *Caulerpa cupressoides* and were named CCB-F0.3; CCB-F0.5; CCB-F1.0; and CCB-F2.0. These SPs interacted *in vitro* with calcium oxalate crystals, making their surface more negative. They also induced a decrease in the size and an increase in the number of formed crystals. This effect did not depend on the amount of sulfate groups present in SPs. Except for CCB-F2.0, SPs induced the formation of a greater amount of COD crystals compared to the control group, with CCB-F0.3 being the most efficient. This occurred because CCB-F0.3 was distributed in the base:apex:face of the crystal in a ratio of 2:1:1. We believe that this balance/proportion between SPs at the interaction points was crucial to avoid dehydration of the COD crystal to COM, which enhanced their stability.

## Figures and Tables

**Table 1:** Chemical characterization of SPs from *C. cupressoides*.

Polysaccharide	(SO_4_)/Total Sugar (%/%)	Protein (%)
CCB-F0.3	1.10 ± 0.02 ^a^	0.06 ± 0.01 ^a^
CCB-F0.5	0.86 ± 0.03 ^b^	0.05 ± 0.01 ^b^
CCB-F1.0	0.85 ± 0.02 ^b^	0.11 ± 0.02 ^c^
CCB-F2.0	0.72 ± 0.01 ^c^	0.11 ± 0.01 ^c^

^a–c^ Different letters indicate a significant difference (*p* < 0.05) between the SPs of *C. cupressoides* for each analysis performed separately. d-Galactose and sodium sulfate were used as standards for sugar and sulfate quantification, respectively. The concentration of each SP used to perform the iron chelation test was 1.5 mg/mL. Data are expressed as the mean of three determinations ± standard deviation.

**Table 2:** Number and average size of the crystals formed by treatment with sulfated polysaccharides from *C. cupressoides*.

	Total Amount of Crystals (units)	COM (units)	COM Size (µm)	COD (units)	COD Size (µm)
Control CaOx	21 ± 4.4 ^a^	13 ± 4.1 ^a^	11.9 ± 0.16 ^a^	7 ± 2.0 ^a^	12.8 ± 0.89 ^a^
CaOx + CCB-F0.3	257 ± 4.2 ^b^	48 ± 10.9 ^b^	1.7 ± 0.08 ^b^	209 ± 12.3 ^b^	2.55 ± 0.19 ^b^
CaOx + CCB-F0.5	77 ± 8.3 ^c^	20 ± 6.9 ^a^	4.2 ± 0.64 ^c^	58 ± 9.8 ^c^	4.9 ± 0.12 ^c^
CaOx + CCB-F1.0	184 ± 10.8 ^d^	66 ± 9.1 ^b^	4.4 ± 0.29 ^c^	118 ± 9.8 ^d^	4.7 ± 0.79 ^c,d^
CaOx + CCB-F2.0	32 ± 9.4 ^a^	23 ± 7.3 ^a^	4.8 ± 0.96 ^c^	9 ± 3.3 ^a^	6.2 ± 0.83 ^d^

COM, monohydrate calcium oxalate crystals; COD, dihydrate calcium oxalate crystals; COT, trihydrate calcium oxalate crystals. COTs were only found in the control (1 ± 0.9 units). For each analysis performed, different letters (^a–d^) indicate a significant difference (*p* < 0.05) between the crystals formed with absence (Control CaOx) and presence of SPs of *C. cupressoides*.

**Table 3:** Zeta potential of crystals formed in the presence of polysaccharide fractions of *C. cupressoides* seaweed at 25 °C.

	ζ (mV)
Control CaOx	+8.85 ± 3.30 ^a^
CaOx + CCB-F0.3	−25.82 ± 6.36 ^b^
CaOx+ CCB-F0.5	−43.87 ± 8.63 ^c^
CaOx + CCB-F1.0	−51.50 ± 2.14 ^c^
CaOx + CCB-F2.0	−68.70 ± 12.01^d^

For each analysis performed, different letters (^a–d^) indicate a significant difference (*p* < 0.05) between the crystals formed with absence (Control CaOx) and presence of SPs of *C. cupressoides*.
